# *Aspergillus* Serology Predicts Relapse in Chronic Pulmonary Aspergillosis: Implications for Personalised Follow‐Up Strategies

**DOI:** 10.1111/myc.70156

**Published:** 2026-01-30

**Authors:** Annabel Choyce, Stefano Colombo, Cyrin Cyriac, Abdulrazaq Alasfour, Wai Kien Ng, Lauren Amphlett, Chris Kosmidis

**Affiliations:** ^1^ National Aspergillosis Centre, Department of Infectious Diseases Manchester University NHS Foundation Trust Manchester UK; ^2^ Lydia Becker Institute of Immunology and Inflammation, Faculty of Biology, Medicine, and Health, Manchester Academic Health Science Centre The University of Manchester Manchester UK; ^3^ Faculty of Biology, Medicine, and Health, Manchester Academic Health Science Centre The University of Manchester Manchester UK; ^4^ Department of Infectious Diseases Manchester University NHS Foundation Trust Manchester UK; ^5^ Manchester Fungal Infection Group, Faculty of Biology, Medicine, and Health Manchester Academic Health Science Centre, University of Manchester Manchester UK

**Keywords:** *aspergillus*, chronic pulmonary aspergillosis, relapse, risk factors, serology

## Abstract

**Introduction:**

Relapses occur in up to 40% of patients after prolonged courses of antifungal treatment for chronic pulmonary aspergillosis (CPA). The factors predisposing to relapse remain poorly defined.

**Methods:**

We conducted a retrospective study of adults treated for ≥ 6 months with oral azoles for CPA. Patients who completed antifungal therapy and were deemed not to require further treatment were included. Demographic, clinical, radiological and serological data at treatment completion were collected. CPA relapse was defined as symptomatic and radiological deterioration leading to re‐initiation of antifungal therapy. Cox regression and Kaplan–Meier analyses were used to identify predictors of relapse and mortality.

**Results:**

Among 125 patients (56% male; mean age 61 years), median treatment duration was 36 months. Thirty‐two (26%) developed relapse; relapse rate at 1 year was 16%. *Aspergillus* sensitisation (specific IgE > 0.35 IU/mL) and elevated *Aspergillus*‐specific IgG (> 40 mg/L) at treatment completion were independently associated with relapse (*p* < 0.05). No patient with IgG < 40 mg/L relapsed. Underlying lung disease (COPD or prior tuberculosis), extent of radiological involvement, or treatment duration were not significantly associated with relapse or mortality.

**Conclusions:**

*Aspergillus* sensitisation and persistently elevated *Aspergillus*‐specific IgG at the end of antifungal therapy were independent predictors of CPA relapse. These parameters may reflect ongoing fungal airway burden and can help identify patients requiring extended or closer post‐treatment follow‐up. Underlying comorbidities were not associated with relapse risk.

## 1 Introduction

Chronic pulmonary aspergillosis (CPA) is a progressive, indolent fungal infection that requires prolonged antifungal therapy. Standard management typically involves long‐term triazole treatment, as clinical improvement is often slow. Current guidelines recommend at least 6 months of therapy, followed by clinical, radiological and serological reassessment of treatment response [1]. In patients who demonstrate significant improvement, antifungal therapy may be discontinued, whereas others require extended or even indefinite treatment [1]. In patients who deteriorate despite antifungal treatment, emergence of azole resistance, subtherapeutic drug levels or alternative pathology need to be considered.

Despite these prolonged treatment courses, relapse is common, with reported rates ranging from 21% to 39% [2, 3, 4, 5, 6, 7]. Relapse is most often defined as clinical and radiological deterioration after completing at least 6 months of antifungal therapy, although some studies have also incorporated serological or microbiological criteria [6]. Management of relapse is particularly challenging due to the risk of azole resistance, further progression of lung disease and the impact of declining respiratory reserve and frailty. Moreover, relapses may go undetected in patients who are lost to follow‐up once treatment is discontinued.

Although diagnostic and therapeutic strategies for CPA have advanced, the factors that predispose to relapse remain poorly understood. Duration of therapy, baseline disease burden and serological markers have each been proposed as potential contributors, but evidence is limited [3, 4, 5, 6, 7]. Better understanding of the risk factors for relapse could support a more personalised approach to CPA management by identifying patients who may benefit from closer surveillance or extended, potentially lifelong antifungal treatment.

In this study, we evaluate clinical, radiological and serological parameters at the time of treatment completion to identify predictors of relapse in CPA.

## 2 Materials and Methods

### 2.1 Patients

This was a retrospective study of adult patients who received at least 6 months of azole treatment for CPA at the National Aspergillosis Centre, Manchester.

Patients who finished a course of antifungal treatment of 6 months or longer and were deemed by the treating clinician not to require further antifungal treatment for CPA at that point were eligible. Patients who were switched from one antifungal agent to another, due to intolerance or resistance, were included as long as the treatment gap between the antifungal agents was < 6 weeks. This was allowed in order to account for the common real‐world situation when treatment is halted until side effects of treatment such as raised liver function tests return to baseline before a different azole is introduced as part of the same treatment course.

### 2.2 Data Collection and Definitions

The information collected involved demographics, pulmonary and other co‐morbidities, antifungal treatment (start and stop date), extent of disease on imaging at the time of treatment completion (unilateral vs. bilateral, number of cavities, presence of aspergillomas) and *Aspergillus* serology. As this was a real‐world study, the CT scan closest to the treatment completion date was selected, provided it was performed within 3 months of the date of treatment completion. Serum concentrations of *Aspergillus* IgG, total IgE and *Aspergillus*‐specific IgE were recorded if they were available within 3 months of the date of treatment completion. Patients were defined as *Aspergillus*‐sensitised when Aspergillus‐specific serum IgE concentration was > 0.35 IU/mL. *Aspergillus*‐specific IgG and IgE were measured using the ImmunoCAP assay (Thermo Fisher Scientific).

CPA relapse was defined as: (a) symptoms suggestive of CPA as assessed in contemporaneous clinical notes, prompting a chest CT scan, (b) CT scan findings suggestive of CPA relapse, (c) reasonable exclusion of alternative diagnoses by the clinical team and (d) a clinical decision to re‐initiate antifungal treatment. CT scan features deemed suggestive of CPA relapse included a new or enlarging aspergilloma, a new or enlarging cavity, increased cavity wall thickness, increased pleural thickening and increasing consolidation surrounding a preexisting cavity.

The date of documented relapse or the time of the last clinical follow up or death were recorded.

This retrospective study analysed anonymised data collected during routine clinical care. No additional procedures were performed, and all data were anonymised prior to analysis; formal research ethics committee approval and individual patient consent were not required.

### 2.3 Statistical Analysis

Statistical analysis was performed using R version 4.4.1. For analysis of risk factors associated with mortality the period of assessment was defined as from the date of initial diagnosis of CPA to the date of outcome (death or censor). For analysis of relapse‐free survival the period of assessment was defined as from the date of treatment ending to the date of relapse assessment. Clinical characteristics (Table 1) present in > 10% of the cohort were selected for risk‐factor analysis. Multivariate Cox proportional hazards regression models were fitted to the data using the ‘survival’ package, version 3.6‐4. Kaplan–Meier plots were generated using the ‘survival’ package. Univariate statistical comparisons were calculated via log‐rank test using the ‘survminer’ package, version 0.4.9.

**TABLE 1:** Clinical characteristics of 125 chronic pulmonary aspergillosis patients.

	*N* (%)
Underlying disease predisposing to CPA	
COPD	55 (44)
Bronchiectasis	47 (38)
Asthma	24 (19)
Prior tuberculosis	23 (18)
Prior pneumonia	21 (17)
Prior chest surgery	16 (13)
Prior cancer	13 (10)
Sarcoid	10 (8)
Pneumothorax	10 (8)
ABPA	8 (6)
Prior NTM‐PD	8 (6)
Rheumatoid arthritis	8 (6)
Interstitial lung disease	4 (3)
Ankylosing spondylitis	1 (1)
Other conditions	
Diabetes	9 (7)
Heart failure	8 (6)
Renal failure	3 (2)
First antifungal agent	
Voriconazole	57 (46)
Itraconazole	51 (41)
Posaconazole	15 (12)
Isavuconazole	2 (2)
*Aspergillus* IgG at treatment completion, median (range), mg/L	84 (7–1187)
Total IgE, median (range), IU/mL	89 (2–13285)
*Aspergillus‐*sensitised (*Aspergillus* IgE > 0.35 IU/mL)^a^	50 (48%)
Features on CT scan at treatment completion	47 (38)
Bilateral disease	
Presence of cavity	
None	12 (10)
One	70 (56)
More than one	43 (34)
Presence of aspergilloma	
None	65 (52)
One	48 (38)
More than one	12 (10)

Abbreviations: ABPA, allergic bronchopulmonary aspergillosis; COPD, chronic obstructive pulmonary disease; CPA, chronic pulmonary aspergillosis; NTM‐PD, non‐tuberculous mycobacterial pulmonary disease.

^a^ Information available in 105 patients.

## 3 Results

125 patients completed at least 6 months of azole treatment for CPA and were followed for relapse. Seventy (56%) were male and mean age at completion of treatment was 61 years (range 25–83). Median duration of antifungal treatment was 36 months (range 6–216 months). Patient characteristics are presented in Table 1.

**FIGURE 1:**
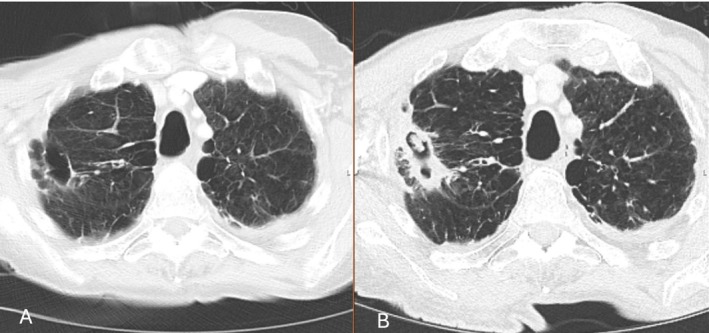
Follow‐up chest imaging of a 75‐year‐old man with COPD. (A) CT scan at end of antifungal treatment. (B) One year after stopping antifungal therapy, the scan shows reappearance of a fungal ball within the cavity, thickening of the cavity wall, and adjacent pulmonary consolidation.

**FIGURE 2:**
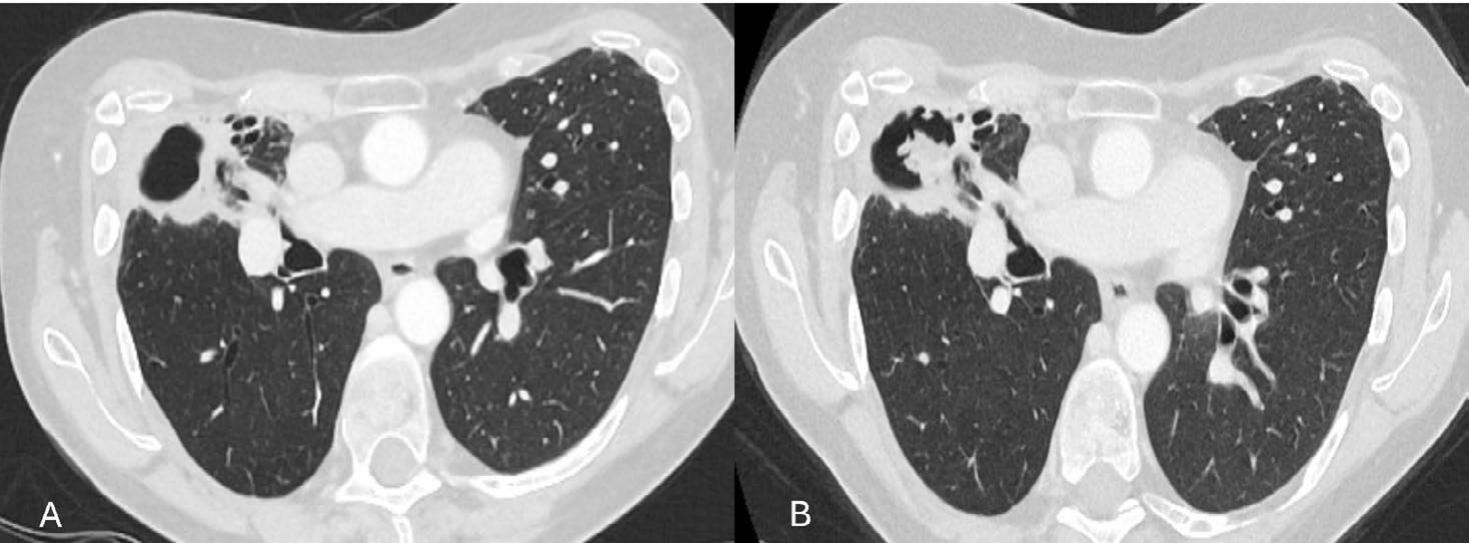
56‐year‐old woman with a past medical history of asthma, bronchiectasis and previous thoracic surgery for pectus excavatum. She was previously treated with voriconazole for 2 years and discontinued therapy due to clinical stability. (A) CT scan at end of treatment. (B) One year after stopping antifungal treatment, she reported worsening dyspnoea and a productive cough. A CT scan demonstrates a new fungal ball within the right upper lobe cavity.

Thirty‐two (26%) patients had a relapse. The radiological features of relapse are described in Table 2. An example of a case of relapse based on CT imaging, which shows a new cavity with aspergilloma formation is shown in Figure 1. A further case with a new aspergilloma within a preexisting cavity is shown in Figure 2. Among 100 patients who had follow up data for more than 12 months, 16 (16%) had a relapse within a year after completing the antifungal treatment. The characteristics of patients with and without a relapse are presented in Table 3. Patients with prior TB had a median *Aspergillus* IgG of 121.5 mg/L compared to 82 mg/L for those without TB (*p* = 0.090). There were no significant differences in the rates of *Aspergillus* sensitisation between patients with prior TB or COPD.

**TABLE 2:** Radiological features in 32 patients with CPA relapse.

Radiological change	*N* (%)
Increase in cavity wall thickness	10 (31)
Enlargement of existing cavity	9 (28)
Progression of peri‐cavitary consolidation	9 (28)
Increase in aspergilloma size	8 (25)
New aspergilloma	7 (22)
New cavity	4 (13)
Increase in pleural thickening	2 (6)
New nodules	2 (6)
Progressive fibrosis	2 (6)
Enlarging nodules	1 (3)
New fluid level	1 (3)
Invasion of pulmonary artery	1 (3)

**TABLE 3:** Characteristics of patients with and without a relapse.

Characteristics	Relapse (*n* = 32)	No relapse (*n* = 93)	*p*	OR (95% CI)
Age, mean ± SD	59.8 ± 11.8	61.4 ± 11.7	0.494	
Male	16 (23)	54 (77)	0.536	0.72 (0.32–1.62)
Female	16 (29)	39 (71)		
COPD	14 (26)	41 (75)	0.974	0.99 (0.44–2.22)
Prior tuberculosis	9 (39)	14 (61)	0.116	2.21 (0.85–5.76)
Asthma	7 (29)	17 (71)	0.795	1.25 (0.47–3.37)
Bronchiectasis	10 (21)	37 (79)	0.526	0.69 (0.29–1.62)
Prior pneumonia	6 (29)	15 (71)	0.786	1.2 (0.42–3.41)
Aspergillus IgG at completion, mg/L, median (IQR)	101 (89)	76 (86)	**0.011**	
*Aspergillus* IgG at completion, mg/L				
< 40	0 (0)	21 (100)	**< 0.001**	
> 40	31 (32)	65 (68)		
> 60	25 (32)	53 (68)	0.075	2.59 (0.96–6.99)
> 100	16 (35)	30 (65)	0.134	1.99 (0.87–4.58)
*Aspergillus* sensitisation (*Aspergillus* IgE > 0.35IU/mL)	18 (36)	32 (64)	**0.013**	3.3 (1.28–8.51)
Total IgE, IU/mL, median (IQR)	172 (661)	71 (275)	0.083	
Presence of aspergilloma on CT scan	14 (24)	45 (76)	0.684	0.81 (0.36–1.82)
Bilateral disease on CT scan	13 (28)	34 (72)	0.833	1.17 (0.51–2.66)
Cavity on CT scan				
No cavity	1 (8)	11 (92)	0.225	
One cavity	17 (25)	52 (75)		
More than one cavity	14 (33)	29 (67)		
Treatment duration				
12 months or more	26 (26)	75 (74)	0.940	1.04 (0.37–2.90)
< 12 months	5 (25)	18 (75)		
Duration of treatment, months, median	39.5	35	0.903	
First treatment				
Itraconazole	15 (29)	36 (71)	0.724	
Voriconazole	13 (23)	44 (77)		

*Note:* Figures in Table are *n* (%) unless otherwise specified. *p* values shown in bold are considered statistically significant.

Abbreviation: COPD, chronic obstructive pulmonary disease.

**FIGURE 3:**
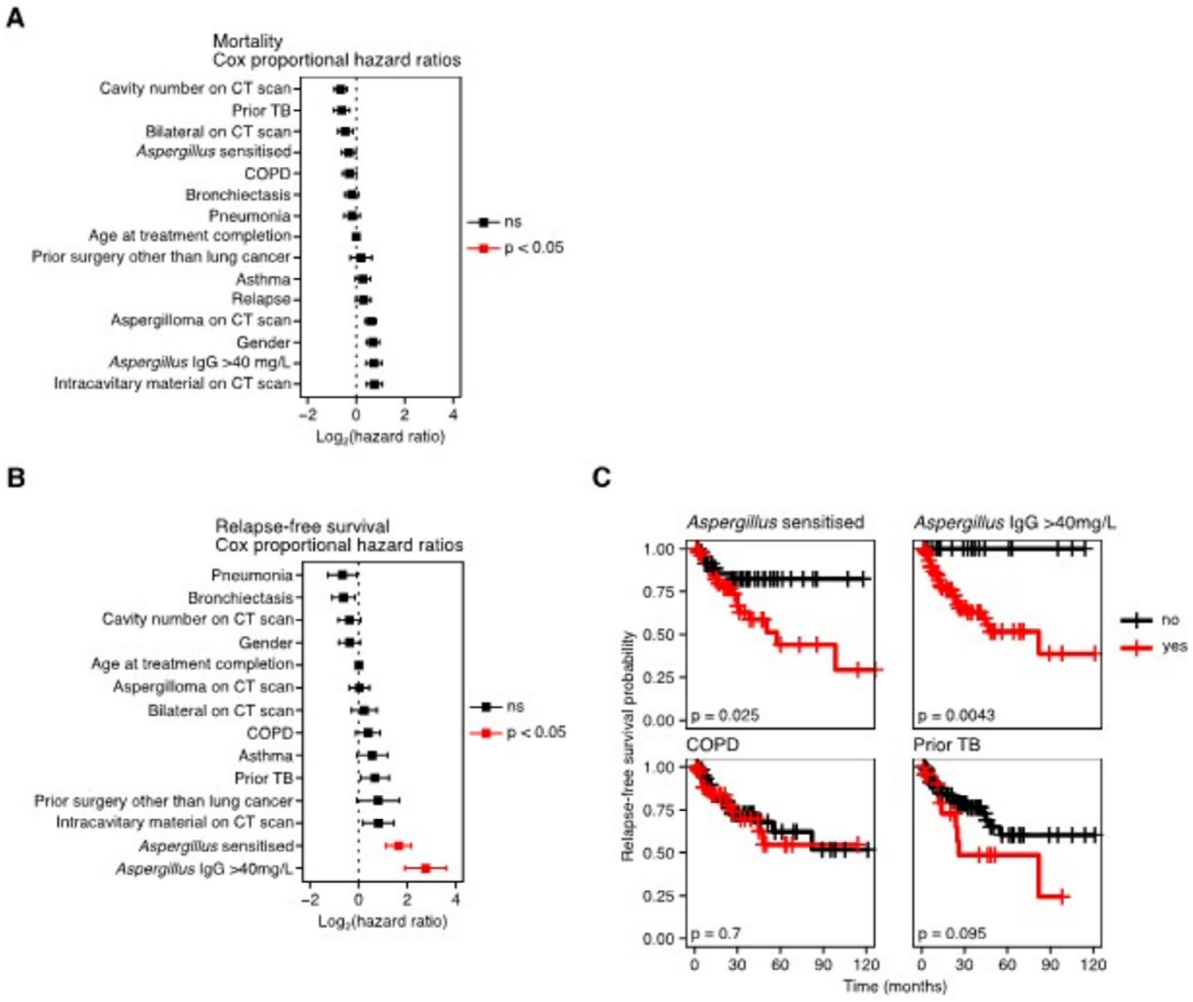
Anti‐*Aspergillus* serum IgE and IgG is associated with relapse. (A, B) Dot plots representing the results of a multi‐variate Cox regression analyses of (A) risk factors for mortality in CPA and (B) risk factors for relapse. Points indicate Log_2_ transformed hazard ratios. Error bars indicate the standard error. Black points indicate non‐statistically significant factors. Red points indicate risk factors where *p* < 0.05 as calculated by Cox proportional hazards regression. (C) Kaplan–Meier curves of selected risk factors for relapse‐free survival. Vertical lines indicate where patients were censored. Black lines indicate where the risk factor was not present. Red lines indicate presence of the risk factor. *p* values were calculated by univariate log‐rank test.

Twenty‐one patients (17%) were dead at the time of the last follow up. Cox regression analysis of risk factors associated with mortality did not identify any variables to be significantly associated with mortality (Figure 3A). However, Cox regression analysis of the risk factors associated with relapse identified *Aspergillus*‐sensitization and an anti‐*Aspergillus* serum IgG concentration > 40 mg/L to be associated with a greater probability of relapse (Figure 3B). This was confirmed by Kaplan–Meier analysis with univariate log‐rank testing (Figure 3C). Notably, COPD and a prior history of TB were not significantly associated with an increased probability of relapse (Figure 3C).

## 4 Discussion

In this retrospective analysis we assessed factors associated with post‐treatment relapse in CPA. Our results demonstrate that the presence of *Aspergillus* sensitization and of a higher *Aspergillus*‐specific IgG titre at completion of antifungal treatment were independently associated with the risk of subsequent relapse. Therefore, we propose that these markers can be used to identify patients who would benefit from a more frequent or more prolonged follow‐up after treatment. In contrast, we did not find a statistically significant link between relapse and underlying lung conditions such as COPD or prior TB. This suggests that the fungal burden in the airways may be a more important determinant of the risk of CPA relapse than the underlying medical background of the host. However, we did observe a trend for more frequent relapse in patients with prior TB. As only 23 of the patients included in this study had a prior history of TB (9 of whom relapsed) it is possible that our analysis of this co‐morbidity was underpowered and further analysis with a larger sample size may identify a significant association between this risk factor and relapse.

The *Aspergillus*‐specific IgG titre is used both in diagnosis and in the monitoring of CPA on and off treatment. While it is expected and desirable to most clinicians that the titre should reduce on treatment, there is no universally accepted practice on its interpretation to guide treatment decisions. In a consensus statement by CPAnet, a clinical research group, it was not recommended that *Aspergillus*‐specific IgG be used to assess treatment response [8]. Furthermore, it is not currently used as a test of cure or as a prognostic marker. Occasionally, the titre falls below the laboratory determined cutoff (< 40 mg/L with the ImmunoCAP assay in our laboratory) which may provide reassurance that the burden of disease is so low that treatment can be safely discontinued. We found that no patient with a titre < 40 mg/L after at least 6 months of antifungal treatment had a relapse, supporting this assumption. Therefore, a higher *Aspergillus* IgG level at treatment completion may necessitate the need for a closer follow up for relapse. However, the relationship between airway fungal burden and *Aspergillus*‐specific IgG titres is not defined. The *Aspergillus*‐specific IgG titre may reflect the humoral response maintained by long‐lived plasma cells, or may fluctuate more short‐term based on ongoing antigenic stimulation in the airways. More likely, both mechanisms may contribute: *Aspergillus‐*specific IgG tends to fall with antifungal treatment, suggesting a response to the reducing fungal airway burden, whereas in most cases an elevated titre remains even after improvement of symptoms and cessation of antifungal therapy, suggesting long‐term memory.

*Aspergillus* sensitisation is common in CPA and seen in almost half of our cohort, although very few had a diagnosis of allergic bronchopulmonary aspergillosis (ABPA). *Aspergillus* sensitisation appears to be a feature of CPA independent of the presence of chronic airways disease, as Sehgal et al. [5] reported it in 40% of patients in their CPA cohort after excluding all patients with asthma and COPD. We found that *Aspergillus*‐sensitised patients were 3 times more likely to develop CPA relapse compared to the non‐*Aspergillus‐*sensitised. Sehgal et al. [5] also reported an association between *Aspergillus* sensitisation and relapse. This may be due to an increased predisposition in these patients for recurrent colonisation with *Aspergillus* and inability to effectively clear it, triggering an exaggerated Th2 response which may manifest as progressive symptoms and radiological findings. Such patients should receive closer follow up for relapse.

Unlike other studies, we did not find an association between the extent of disease and relapse. In a previous study, Bongomin et al. [6] identified bilateral disease as a risk factor for relapse. However, in that study not all patients had radiological confirmation or relapse. Similarly, Koyama et al. [7] identified multilobal involvement as linked with relapse. However that study used precipitin positivity rather than a specific IgG titre with one of the newer immunoassays and did not evaluate *Aspergillus* sensitisation.

Patients with CPA may have a number of different underlying diseases which in theory could affect the risk of relapse, such as COPD or asthma, possibly due to the use of inhaled or oral steroids. Neither we nor others have demonstrated significantly higher relapse rates according to a specific underlying disease. Interestingly, patients with prior TB exhibited a trend for higher relapse rates, although this did not reach statistical significance. This may be linked with the higher *Aspergillus* IgG observed in these patients on treatment completion compared to those without a history of TB. It is possible that the cavitary changes caused by prior TB may be more prone to fungal colonisation compared to cavities of a different causation.

We did not find an association between treatment duration and risk of relapse. However, we acknowledge that this study was not designed to assess the impact of treatment duration on the relapse risk, as a RCT would be required to adequately address this question. Sehgal et al. [4] showed that patients who were randomised to receive 12 months of treatment had fewer relapses within the follow up period. In this study, patients received treatment for longer periods compared to other reported cohorts, reflecting different treatment practices, most likely due to concern by the treating clinician about the relapse risk. Despite these more prolonged regimens, 1 in 4 patients developed relapse, similar to what has been reported in cohorts treated for shorter periods [2, 3, 4, 5, 6, 7]. Therefore, it is possible that long‐term azole treatments may still not reduce the risk of relapse in patients with risk factors such as *Aspergillus* sensitisation or a high *Aspergillus* IgG. Different approaches like combination antifungal therapies should be explored in clinical trials for such patients.

In conclusion, we find that *Aspergillus* sensitisation and a higher *Aspergillus*‐specific serum IgG concentration on completion of treatment were predictive of relapse in CPA, whereas underlying conditions or extent of disease on imaging were not. We suggest that patients with these serological parameters be followed closely after treatment completion and counselled about the risk of relapse to inform decisions about therapy discontinuation.

## Author Contributions

**Annabel Choyce:** investigation, methodology, validation, writing – original draft. **Stefano Colombo:** methodology, software, formal analysis, writing – original draft. **Cyrin Cyriac:** investigation, data curation, visualisation. **Abdulrazaq Alasfour:** investigation, data curation, visualisation. **Wai Kien Ng:** investigation, data curation, visualisation. **Lauren Amphlett:** project administration, resources, visualisation. **Chris Kosmidis:** conceptualization, writing – original draft, writing – review and editing, formal analysis, supervision, methodology.

## Funding

The authors have nothing to report.

## Conflicts of Interest

Chris Kosmidis has received speaker's fees from Pfizer and consultation fees from Mundipharma and Shionogi Europe. The other authors declare no conflicts of interest.

## Data Availability

The data that support the findings of this study are available from the corresponding author upon reasonable request.
